# Seasonal space use of gray wolves is concurrent with primary prey

**DOI:** 10.1007/s00442-026-05907-4

**Published:** 2026-05-25

**Authors:** Nathaniel H. Wehr, Seth A. Moore, Mark C. Romanski, Edmund J. Isaac, Kenneth F. Kellner, Todd M. Kautz, Joshua J. Millspaugh, Jerrold L. Belant

**Affiliations:** 1 Department of Fisheries and Wildlife, Michigan State University, East Lansing, MI USA; 2 Department of Biology and Environment, Grand Portage Band of Lake Superior Chippewa, Grand Portage, MN USA; 3 National Park Service, Isle Royale National Park, Houghton, MI USA; 4 Wildlife Biology Program, Department of Ecosystem and Conservation Sciences, W.A. Franke College of Forestry and Conservation, University of Montana, Missoula, MT USA; 5 Pennsylvania Cooperative Fish and Wildlife Research Unit, The Pennsylvania State University, University Park, Pennsylvania, USA; 6 Minnesota Department of Natural Resources, Grand Marais, MN USA; 7 State University of New York Research Foundation, Albany, NY USA; 8 Department of Environmental Biology, State University of New York College of Environmental Science and Forestry, Syracuse, NY USA

**Keywords:** Ecological seasonality, Moose, Predator–prey, Resource selection, White-tailed deer

## Abstract

Ecological seasonality describes the dynamic adaptation of species to changes in the biotic community and suggests predators should alter space use synchronously with seasonal prey availability. We assessed seasonal space use states (i.e., periods of similar movement and resource use) of mainland gray wolves (*Canis lupus*), moose (*Alces alces*), and white-tailed deer (*Odocoileus virginianus*) on the Grand Portage Indian Reservation, Minnesota, USA and island wolves and moose on Isle Royale National Park, Michigan, USA. We predicted mainland wolves would alter space use states (i.e., ecological seasons) corresponding to sympatric deer migration but island wolves would not, corresponding to their primary prey, non-migratory moose. We also predicted parturition would influence seasonal space use states in both systems. We used GPS collar locations recorded during 2008–2021, cluster analyses to temporally define space use states, and weighted resource selection functions to assess habitat selection during seasonal space use states. Mainland wolves exhibited two seasonal space use states with transitions between seasons coinciding with deer migration, and habitat selection varied among space use states for mainland wolves and their prey. Island wolves and moose did not alter their space use states seasonally, and their habitat selection differed from mainland populations. Parturition did not have a clear influence on seasonal space use. Our results suggest ecological seasonality is moderated by migration and linked to inter- and intra-guild species interactions via seasonal space use dynamics. These findings expand understanding of ecological seasonality at the community level and support the importance of multi-species management decisions.

## Introduction

The Grand Portage Band of Lake Superior Chippewa is a federally recognized sovereign nation of Anishinaabe people whose home is the Grand Portage Indian Reservation, Minnesota, USA (Gichi Onigaming). Tribal bandmembers exercise their usufructuary rights to food sovereignty through subsistence hunting, fishing, and gathering throughout the 1854 Ceded Territory (Thompson 2020). Isle Royale National Park, Michigan, USA (Minong) is a Traditional Cultural Property of the Grand Portage Band. Moose (mooz; *Alces alces*) and white-tailed deer (waawaashkeshi; *Odocoileus virginianus*) are primary subsistence species of Anishinaabe people in the 1854 Ceded Territory. Gray wolves (ma’iingan; *Canis lupus*) are kin to the Anishinaabe people and important to their seventh-generation approach to ecosystem stewardship (Gilbert et al. 2022). The Grand Portage Band conducts predator–prey research to improve their understanding of ecosystem health, which set the context for this study.

Ecological seasonality is based on animal ecology and influenced by biotic (e.g., available forage, reproductive cycles) and abiotic (e.g., weather) factors, as opposed to calendar-derived seasons of fixed duration, such as solstices, or seasons determined using a single variable such as migration timing (Basille et al. 2013). Defined in the context of ecological seasonality, seasonal space use states are time periods identified by population-level space use (i.e., movements and resource selection) resulting from species-specific responses to changing biotic and abiotic conditions. In the context of predator–prey theory (Lack 1954; Werner and Hall 1974; Mitchell and Lima 2002), predators should alter space use states across ecological seasons corresponding with prey community changes resulting from seasonal forage availability and migration (Furey et al. 2018). For example, lions (*Panthera leo*) demonstrated seasonal space use state changes between wet and dry seasons corresponding to spatial concentrations of prey around seasonal water sources (Schooler et al. 2022). Improved insights into ecological seasons can further understanding of community ecology theory and refine data collection and analysis in the context of animal behavior (Basille et al. 2013).

Habitat selection, an important component of space use, is influenced by cost–benefit tradeoffs that maximize fitness (Mayor et al. 2009). For prey, optimal habitat selection depends on maximizing nutritional intake and minimizing predation risk (Brown et al. 1999), while predators depend on matching prey habitat selection across space and time (Mitchell and Lima 2002). Predator adaptations may therefore correspond to seasonal variability in prey availability (i.e., the relative abundance and vulnerability of prey species) (Lack 1954; Werner and Hall 1974; Tschanz et al. 2007). Eurasian otters (*Lutra lutra*), for example, switched from greater amphibian consumption to greater eel (*Anguilla anguilla*) consumption during April–May corresponding to amphibians’ spring migration away from aquatic breeding grounds and juvenile eels’ spring migration into estuaries (Moorhouse-Gann et al. 2020).

Though optimal space use among predators is primarily determined by prey consumption, reproduction also influences space use. Baleen whales (parvorder Mysticeti) migrated to avoid seasonal orca (*Orcinus orca*) predation of calves (Corkeron and Connor 1999). Male bottlenose dolphins (*Tursiops aduncus*) selected prey-rich offshore embankments except when females entered estrus; male dolphins then switched to sheltered habitats used by females forfeiting higher prey abundances (O’Brien et al. 2020). Steller sea lion (*Eumetopias jubatas*) (Sinclair and Zeppelin 2002) and cougar (*Puma concolor*) (Smereka et al. 2020) females selected habitats decreasing predation of their dependent young in lieu of habitats with higher prey concentrations, conceding prey encounters. These space use changes suggest a necessary balance between food consumption and sexual reproduction, which should inherently influence ecological seasonality.

Gray wolf space use is similarly influenced by prey availability and reproduction. Where wolves’ primary prey are migratory, they alter space use to match prey distributions. Wolves migrated between winter and summer ranges following caribou (*Rangifer tarandus*) migrations (Walton et al. 2001) and followed elevation-mediated habitat selection by moose and caribou associated with snow-depth and forage (Anderson 2014). In contrast, wolves pursuing non-migratory black-tailed deer (*Odocoileus hemionus sitkensis*) did not alter space use seasonally (Roffler et al. 2018). Wolves also switch between prey seasonally according to availability, such as consuming more beaver (*Castor canadensis*) and caribou in summer when these species are more vulnerable (Latham et al. 2013). Reproduction also influences wolf space use; wolves concentrate their movements around den sites following parturition (Benson et al. 2015; Roffler and Gregovich 2018).

**Fig. 1:**
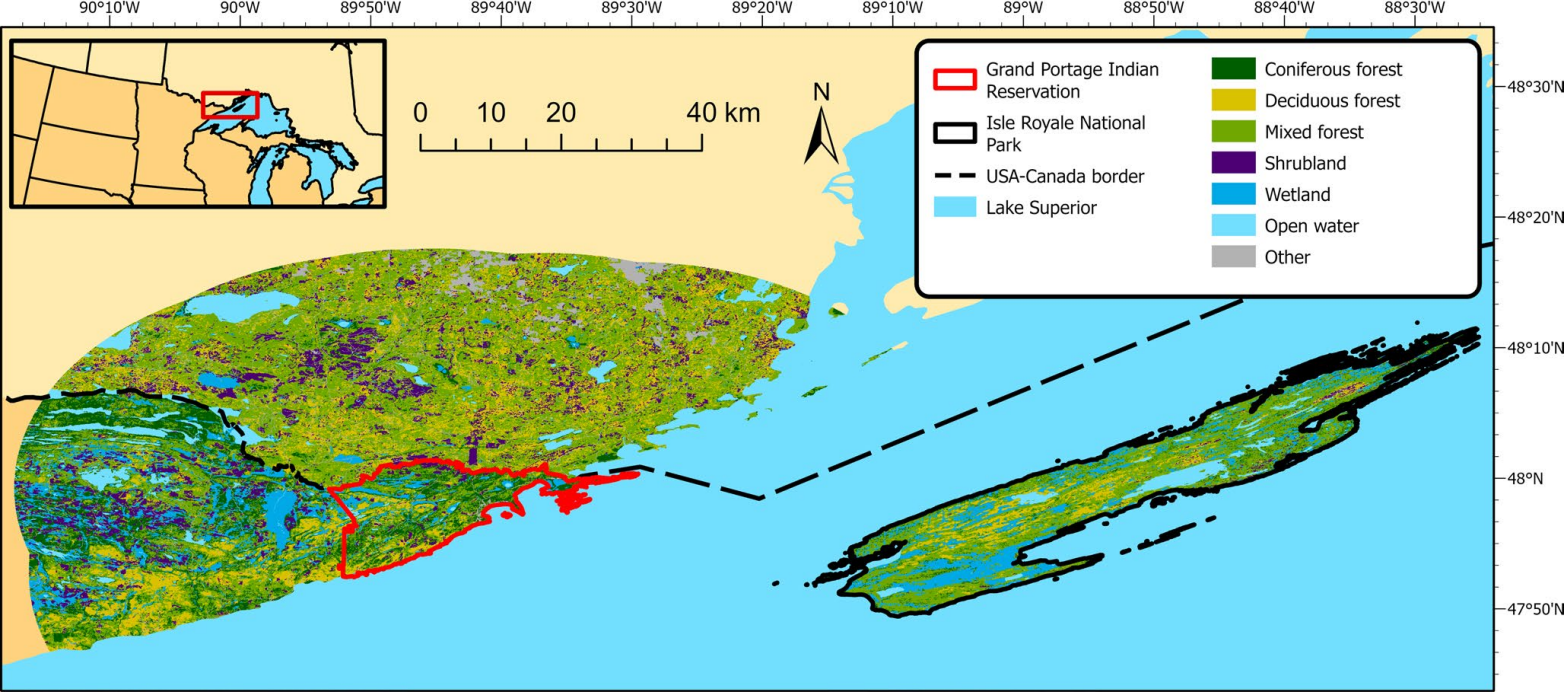
Study area including the mainland ecosystem on and near the Grand Portage Indian Reservation, Minnesota, USA and island ecosystem on Isle Royale National Park, Michigan, USA. Land cover data was obtained from the Commission for Environmental Cooperation 30-m land cover map of North America (CCRS et al. 2020)

We examined gray wolves’ seasonal space use states in two environmentally similar systems with disparate prey communities (Fig. 1) to provide insights into the ecological seasonality concept. In our mainland study system, two-thirds of deer exhibit synchronous spring (April) migration to summer ranges north of the Grand Portage Indian Reservation and asynchronous fall (October–November) migration back to their winter ranges (Wehr et al. 2024b). The remaining third of deer maintain a single home range on or near the Grand Portage Indian Reservation year round (Wehr et al. 2024b). Mainland moose exhibit nomadic (55%), resident (35%), and migratory (10%) seasonal movement strategies (Wehr et al. 2024b). Mainland wolves exhibit prey switching, but not range-shifts, in response to seasonal deer migration (Wehr et al. 2024b), with deer experiencing increased mortality during fall migration due to wolves (Wehr et al. 2024a). On our island study area, deer are not present (Wehr et al. 2023) and moose are considered non-migratory, though they exhibit some seasonal differences in the portions of the island in which they are most observed (Stephens and Peterson 1984; Tischler et al. 2022). We predicted seasonal space use alterations by wolves would coincide with seasonal deer migration on the mainland due to altered prey availability but remain largely unchanged on the island where primary prey are non-migratory. We also predicted wolf space use would be limited by den site fidelity during pup-rearing as a necessary response to parturition but would otherwise reflect habitats used for optimal foraging of primary prey.

## Materials and methods

### Study area

We conducted our study on and near the Grand Portage Indian Reservation (47.9614° N, 89.7594° W) and Isle Royale National Park (47.9959° N, 88.9093° W) (Fig. 1). The Grand Portage study area comprised about 1,200 km^2^ including mainland areas ≤ 30 km from the Grand Portage Indian Reservation (i.e., areas within the migratory range of our study species [Wehr et al. 2024b]) including portions of Ontario, Canada to the north and U.S. federal, state, and private land to the west. This mainland study area is characterized by steep ridges and broad valleys with elevations 183–674 m above sea level (USGS 2020). The mainland area contains 44% mixed forest, 17% deciduous forest, 11% coniferous forest, 9% shrubland, 7% wetland, and 5% open water, with the remainder composed of < 5% each of other habitat types (CCRS et al. 2020). Mean daily temperatures during 2009–2019 ranged from minima of -17.8 ± 3.5 C° (mean ± SD) in January to maxima of 23.3 ± 1.7 C° in July, with 83.8 ± 11.7 cm of rainfall and 150.2 ± 80.8 cm of snowfall annually (NOAA 2024). Wolves on the Grand Portage Indian Reservation represented three packs as well as wolves with no pack fidelity (Wehr 2023). Moose, white-tailed deer, and beaver are the primary prey of gray wolves in this region (Chenaux-Ibrahim 2015). Wolves were legally harvested in Ontario during our study, but legal harvest in Minnesota occurred only during 2012–2014. White-tailed deer and moose were harvested throughout Ontario and Minnesota, but after 2012, only Indigenous bandmembers could harvest moose in Minnesota.

An approximately 534 km^2^ archipelago of over 450 islands, Isle Royale National Park is a United Nations Educational, Scientific and Cultural Organization World Biosphere Reserve in northwestern Lake Superior about 22 km from mainland Minnesota and Ontario. This island study area contains elevations 183–429 m above sea level with rocky terrain, steep parallel ridges, and low-lying areas containing inland lakes (USGS 2020). Land cover of Isle Royale National Park is 49% mixed forest, 23% wetland, 9% deciduous forest, 8% coniferous forest, and 8% open water, with the remainder composed of < 5% each of other habitat types (CCRS et al. 2020). During 2009–2019, Isle Royale National Park had mean January daily minima of -13.4 ± 3.1 C° and mean July daily maxima of 24.2 ± 1.8 C° (NOAA 2024). Wolves on Isle Royale National Park represented 1–2 packs, with as many as half of all wolves not affiliated with a pack (Hoy et al. 2019; Romanski et al. 2020). Moose are the only ungulate on the island and the principal prey of gray wolves along with beaver (Sovie et al. 2023; Wehr et al. 2023). There was no harvest of moose or wolves on Isle Royale National Park.

### Data collection

Mainland data was provided by the Grand Portage Band of Lake Superior Chippewa Department of Biology and Environment. Mainland gray wolves (beginning in 2008), moose (2010), and white-tailed deer (2016) were captured on the Grand Portage Indian Reservation each year. All capture and handling protocols were approved by the Grand Portage Band of Lake Superior Chippewa Tribal Council as well as the Michigan State University institutional animal care and use committee (IACUC; PROTO202200266) and the State University of New York College of Environmental Science and Forestry IACUC (210702). Wolves were captured using foothold traps (checked daily), sedated with ketamine (7.5 mg/kg) and xylazine (1.5 mg/kg), and fitted with GPS collars (Oliveira-Santos et al. 2021). Captured wolves were typically sedated for < 30 min. During sedation, wolves were examined for injuries but only minor issues were observed (e.g., swelling of captured legs). Juvenile black bears (*Ursus americanus*) were infrequently captured in foothold traps targeting wolves. Bear handling protocols followed ongoing research led by the Grand Portage Band of Lake Superior Chippewa (e.g., Moore et al. 2024) and were included in appropriate IACUC protocols. Moose were captured by aerially darting from helicopters with sedation via intramuscular darts using thiafentanil (10 mg) and xylazine (30 mg) (Ienello et al. 2025). Captured moose were fitted with GPS collars and received a physical assessment of body condition alongside sample collections, showing minimal negative effects from the capture process (Ienello et al. 2025). White-tailed deer were captured using corn-baited clover traps (checked daily) and fitted with GPS collars (Oliveira-Santos et al. 2021). Deer were not sedated so long as handling protocols could be completed safely and were typically handled for < 5 min. If necessary, deer were sedated using ketamine hydrochloride (3.5–7.5 mg/kg) and xylazine (1–3 mg/kg) (Oliveira-Santos et al. 2021).

Our access to data collected on Isle Royale was approved by the National Park Service (Permit: ISRO-2018-SCI-0026). All animal capture and handling performed on Isle Royale was conducted in accordance with relevant National Park Service and Grand Portage Band of Lake Superior Chippewa protocols and regulations. From September 2018 to September 2019, 19 wolves were captured in coastal and island habitats of the Lake Superior watershed (Romanski et al. 2020). Captured wolves were examined by a wildlife veterinarian, fitted with GPS collars, and translocated to Isle Royale National Park where two wolves were already present (Orning et al. 2020; Romanski et al. 2020). Five additional wolves born on the island were captured using foothold traps in May 2021 following the same protocols as applied in Grand Portage. Moose on Isle Royale were captured in February 2019 and March 2020 using aerial darting following the same protocols as applied in Grand Portage.

We used GPS-monitoring data collected from captured individuals beginning with captures in 2008 through 31 December 2021. GPS collars included species-appropriate models produced by Vectronic Aerospace (Berlin, Germany). Most collar data was obtained via satellite when collars were active; remaining data were downloaded from collars upon retrieval. Minimum relocation intervals of gray wolf and white-tailed deer collars were 3.25–4.5 h (based on intended collar longevity), and minimum relocation intervals of moose collars were 0.25–4 h (based on time of year). We excluded location data within seven days of capture (including translocation) or mortality to account for capture related movements and, in some cases, our inability to determine exact mortality dates (Northrup et al. 2014). We included all available collar data from Isle Royale following translocation events as instability resulting from intraspecific strife was short-lived and territorial behaviors were exhibited among individuals within the small population (Hoy et al. 2019; Romanski et al. 2020).

We produced rasters of land cover, aspect, slope, and distance to Lake Superior, roads (mainland only), and trails (island only) using ArcGIS Pro (v3.0.3, Esri, Redlands, California, USA). We used the Commission for Environmental Cooperation 30-m resolution land cover map of North America (CCRS et al. 2020). We considered agriculture and grassland part of the shrubland category for our analyses because these three categories were frequently misidentified as one another and composed < 2% of land cover. We derived aspect and slope from the United States Geological Survey 1 arc-second (about 30-m resolution) digital elevation models (USGS 2020). We used the *Euclidian distance* tool to calculate the nearest distance from each 30-m cell to Lake Superior as defined by the National Oceanic and Atmospheric Administration (NOAA) digital shoreline (NOAA 2000), to roads demarcated by the United States Census Bureau or Ontario Road Network (USCB 2021; OMNRF 2022), and to trails using a National Park Service database. Distance to Lake Superior was used as a covariate in our models to account for the use of lakeshore habitats as a predation refuge in both study systems (Stephens and Peterson 1984; Wehr et al. 2024b), and distances to roads and trails were similarly used to account for a potential predator refuge or trap in the context of human shield effects and wolf movements (Berger 2007; Darlington et al. 2022).

We obtained weather data from the NOAA Climate Data Online tool as the daily average of data recorded from weather stations in Cook County, Minnesota, USA during 2008–2021 (NOAA 2024). We calculated average daily change in growing degree days (ΔGDD) from mean daily temperatures using a latitudinal correction (van Wijk et al. 2012). In our study area, this resulted in a plant growth threshold of approximately 1 C°.

### Ecological seasonality

We determined the occurrence and timing of seasonal space use states for each population using cluster analyses in R (v4.2.1) using the *clusterSim* and *fpc* packages (Basille et al. 2013; Hennig 2020; Walesiak and Dudek 2021; R Core Team 2025). Cluster analyses group data points with similar characteristics and can be applied to any vector describing a study object (Frades and Matthiesen 2010). We conducted cluster analyses using year-round data and did not limit clusters to specific timeframes. We resampled collar locations to 12 ± 2 h relocation intervals to reduce biases resulting from relocation intervals varying across individuals and seasons using the *amt* package (Signer et al. 2019). We calculated step-length and turning angle for each GPS collar location. Step-length was the distance between the prior and current locations, and turning angle was the angle formed by the prior, current, and subsequent locations. We extracted landscape covariate values for each location and then calculated the daily population average for each covariate from all available locations for each ordinal day to generate population-level, rather than individual-level, clusters. We assessed normality of daily averages using quantile–quantile plots and made square root or center and scale transformations as necessary (Fox 2015; Kassambra 2023). We checked daily averages of landscape covariates for multicollinearity using Pearson’s product-moment correlation coefficient (*r*), assumed influential when |*r*| ≥ 0.70 (Dormann et al. 2013). These steps output a matrix of daily space use averages for each population comprised of individual movements and habitat use (hereafter, daily space use matrix).

We used the daily space use matrices to conduct our cluster analyses. We first generated heuristic identification of noisy variables (HINoV) models using the *clusterSim* package to determine the inclusion and exclusion of space use metrics (Carmone et al. Jr 1999; Walesiak and Dudek 2021). The HINoV process assesses which variables contain little clustering information so they can be removed to reduce production of misleading results (Carmone Jr et al. 1999). We used one-way analysis of variance to identify variables whose distributions differed (α < 0.05) and removed those variables (Carmone Jr et al. 1999). We determined the number of clusters (*k*) to be considered using the elbow method in the *factoextra* package (Kassambra and Mundt 2020) and the silhouette method via manual programming (de Amorim and Hennig 2015). The elbow and silhouette methods allow for visual determination of *k* (de Amorim and Hennig 2015; Kassambra and Mundt 2020).

We used k-means cluster analyses to determine cluster assignments for each day in our daily space use matrices. Our k-means process assigned each day to a cluster by minimizing the mean squared distance from each day’s space use vector to the center of *k* clusters. We completed our cluster analysis using the *fpc* package with 5,000 bootstrap replicates for each of ten random number seeds for the range of *k* (Hennig 2020). To improve robustness of inference, if clusters from any of the ten seeds were unstable per Jaccard similarity index (γ < 0.75), we considered cluster formation not possible (Hennig 2007). If all ten clusters were stable, we assigned the cluster values from the first seed to each day and transposed daily cluster assignments as the mode of a 5-day moving window centered on each ordinal day (Basille et al. 2013). We calculated transition dates between seasonal space use states (i.e., clusters) as the average date between eight consecutive days of cluster assignments occurring in a new category chronologically and reverse-chronologically rounded to the nearest day. We used this approach to overcome selection biases resulting from moving windows and to account for daily cluster assignments occurring outside final assigned seasonal space use states (Basille et al. 2013). Using the assigned dates and daily space use matrices, we applied principal component analyses to identify differences between clusters (Frades and Matthiesen 2010).

### Resource selection

To more robustly assess space use during seasonal space use states, we used weighted autocorrelated kernel density estimation (wAKDE) for weighted resource selection functions (wRSFs) to characterize habitat use for each population within each seasonal space use state delineated by the cluster analyses (Alston et al. 2022). Using wAKDE accounts for spatial and temporal autocorrelation when generating home range estimates, and using wRSFs accounts for model fits across individuals to generate population-level estimates (Alston et al. 2022). This hierarchical approach accounts for pseudoreplication across individuals and typically generates broader confidence intervals than other RSFs, which produces more robust results (Alston et al. 2022). As such, we relied on our wRSFs to assess habitat selection within space use states rather than the principal component analyses loadings estimated as part of the cluster analyses.

We standardized relocation intervals across populations and seasons by resampling moose collar data at 4-h intervals using the *padr* package (Theon 2022). We used shorter relocation intervals than for our cluster analyses because the hierarchical approach allowed for increased resolution without inducing biases (Alston et al. 2022). We subset location data by population, individual, seasonal space use state, and year resulting in individual-space use states as our sample units. If an individual was monitored during the same seasonal space use state across multiple years, we considered the individual-space use states separately to maximize our sample size and account for interannual variability. We excluded location data collected less than two weeks before or after a transition date to help account for daily cluster assignments occurring outside assigned seasonal space use states (Basille et al. 2013). We excluded individual-space use states with < 50 locations, the approximate monitoring time required by gray wolves to traverse their home ranges several times determined visually using variograms in the *ctmm* package (Fleming and Calabrese 2023).

For each individual-space use state, we generated two wAKDE utilization distributions (UDs; 50% and 95%) using the *ctmm* package (Alston et al. 2022; Fleming and Calabrese 2023). We fit integrated RSFs with autocorrelation-adjusted weights (i.e., wRSFs) to estimate the log-likelihood of use as a continuous Poisson point process for each individual-space use state at core (50% UD) and home range (95% UD) levels (Alston et al. 2022; Fleming and Calabrese 2023). We used landscape variables (i.e., land cover [with open water as the reference category], aspect [cosine-transformed to represent northness], slope, and distance to Lake Superior, roads [mainland only], and trails [island only]) as predictors with no interactions. Open water was used as the reference category because its limited use by all species across all seasons provided stability across individual-space use states. To estimate population-level models, we weighted each individual-space use state’s resource selection parameters by their Akaike information criterion for small samples and then calculated the arithmetic mean of the parameters from all individual-space use state models for a given population (Alston et al. 2022; Fleming and Calabrese 2023). This approach is similar to those used in meta-analyses and accounts for the importance of individual-specific slopes (Viechtbauer 2010; Muff et al. 2020; Gill et al. 2023). We determined continuous variables statistically significant if the 95% confidence intervals did not overlap zero.

## Results

We analyzed five populations: mainland gray wolves (35,670 total GPS collar locations), island wolves (32,044 locations), mainland moose (2,179,502 locations), island moose (959,498 locations), and mainland white-tailed deer (139,634 locations). Among 45 mainland wolves (26 females [58%]; median = 623 locations per individual, range = 7–2,721 locations per individual), six dispersed from the study area and were excluded from analyses. Among 24 island wolves (10 females [42%]; median = 1,415, range = 29–3,137), one wolf dispersed to the mainland outside the study area and was not analyzed. All 135 mainland moose (99 females [73%]; median = 2,541, range = 6–16,042) and 44 island moose (38 females [86%]; median = 15,545, range = 516–50,058) remained in the study area. Two of the 74 deer (52 females [70%]; median = 1,363; range = 6–8,497) dispersed from the study area and were excluded from analyses.

**Fig. 2:**
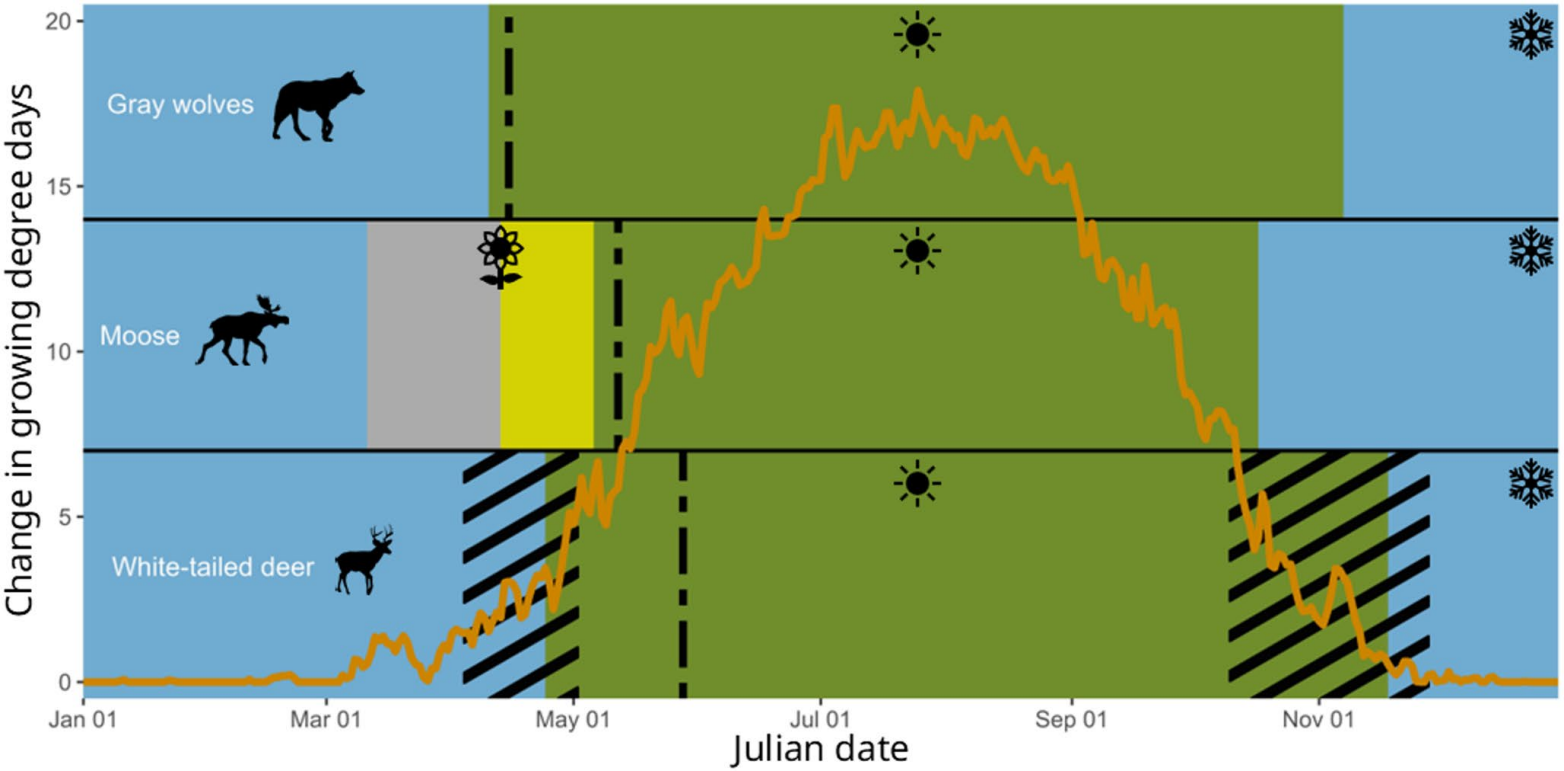
Timing of seasonal space use states for mainland gray wolves (*Canis lupus*), moose (*Alces alces*), and white-tailed deer (*Odocoileus virginianus*) on and near the Grand Portage Indian Reservation, Minnesota, USA, 2010–2021. The orange line represents green-up as the average daily change in growing degree days (ΔGDD). Seasonal space use states are winter (blue, snowflake), summer (green, sun), spring shift (gray, flower), and pre-parturition shift (yellow, flower). Black dashed lines represent median annual parturition dates for each species; black diagonal lines represent spring and fall migration periods for deer. Seasonal space use states are not provided for island wolves and moose on Isle Royale National Park, Michigan, USA, 2018–2021, because space use states did not vary seasonally

**Fig. 3:**
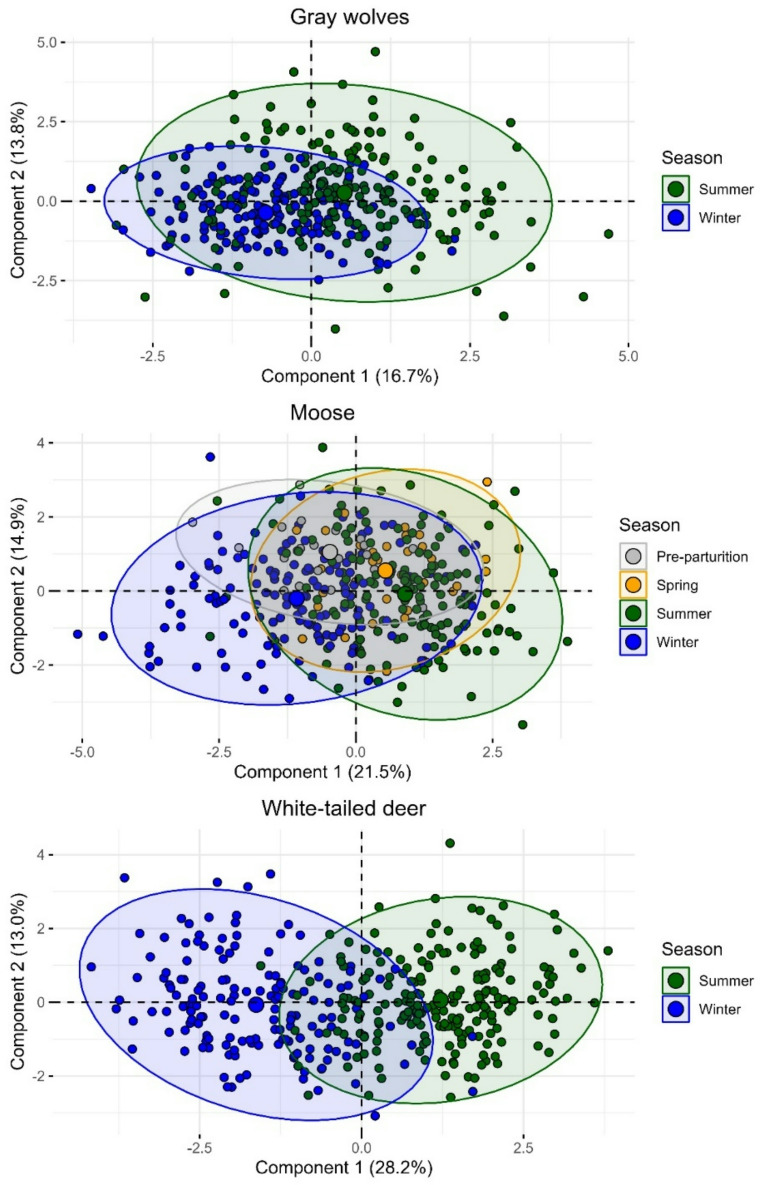
Two-dimensional representation of daily space use variation across seasonal space use states for mainland gray wolves (*Canis lupus*), moose (*Alces alces*), and white-tailed deer (*Odocoileus virginianus*) on and near the Grand Portage Indian Reservation, Minnesota, USA, 2010–2021. Points represent population-level space use for each ordinal date, and shaded polygons represent population-level space use of all ordinal dates comprising a space use state. Component percentages of variation described are included parenthetically. Component loadings are reported in Table 1. Seasonal space use variation is not provided for island wolves and moose on Isle Royale National Park, Michigan, USA, 2018–2021, because space use states did not vary seasonally

All three mainland populations formed stable seasonal space use state clusters (Fig. 2), but island gray wolves and moose did not. Mainland wolves exhibited seasonal space use state clusters during summer (10 April–7 November) and winter (8 November–9 April); the first two principal components described 30.6% of variation between seasons (Fig. 3; Table 1). Mainland moose formed two space use clusters resulting in four seasonal space use states: summer (7 May–17 October), winter (18 October–10 March), spring shift (11 March–13 April), and pre-parturition shift (14 April–6 May); the first two principal components accounted for 36.4% of variation. Mainland white-tailed deer exhibited summer (24 April–18 November) and winter (19 November–23 April) seasonal space use states; the first two principal components described 41.2% of variation between clusters.

**Table 1:** Principle component analysis loadings describing daily space use variation across seasonal space use states for mainland gray wolves (*Canis lupus*), moose (*Alces alces*), and white-tailed deer (*Odocoileus virginianus*) on and near the Grand Portage Indian Reservation, Minnesota, USA, 2010–2021. Loadings represent the direction and strength of a covariate’s influence on space use states in two dimensions (Component 1, Component 2). Differences between seasonal space use states were best described by components 1 and 2 combined for wolves and component 1 only for moose or deer (Fig. 3)

Covariate	Gray wolves		Moose		White-tailed deer	
	Component 1 (16.7%)	Component 2 (13.8%)	Component 1 (21.5%)	Component 2 (14.9%)	Component 1 (28.2%)	Component 2 (13.0%)
Step-length	0.143	0.160	0.304	0.348	0.452	-
Turning angle	-	-	-0.287	-0.131	-	-0.254
Aspect	0.400	0.317	0.276	-0.136	0.298	-
Slope	-0.508	-	-0.361	0.432	-0.116	-0.410
Distance to Lake Superior	0.226	0.462	-0.140	-0.516	0.493	-
Distance to roads	-	-	0.304	-0.275	0.251	-0.425
Coniferous forest	-0.366	-0.248	0.236	0.385	-0.468	0.125
Deciduous forest	0.231	-0.424	-	0.211	0.321	0.224
Mixed forest	-0.326	0.587	-0.243	-	-	-0.571
Shrubland	0.108	-	-0.428	-0.192	0.226	-
Wetland	0.441	-0.212	0.457	0.285	-	0.414

Transitions from winter to spring or summer for mainland populations coincided with white-tailed deer migration and parturition (Fig. 2) as well as green-up and snow melt—ΔGDD and snow depth were negatively correlated (t = -33.33, *p* < 0.01). The gray wolf transition from summer to winter occurred 20 days after the moose transitioned and 12 days before the deer transitioned. The wolf transition from winter to summer occurred 14 days before deer. Because of the two additional transitional seasons in spring, moose transitions from winter to summer were offset from wolves and deer but encompassed wolf and deer seasonal transitions. Deer transitions between seasons occurred near the end of their seasonal migrations.

**Fig. 4:**
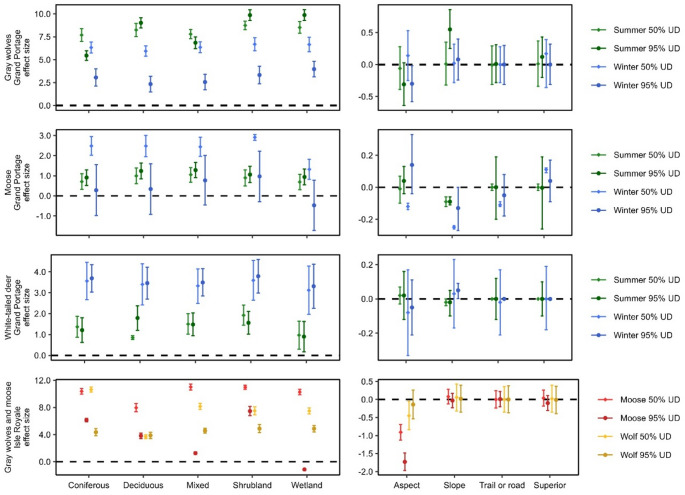
Effect size mean with 95% confidence intervals for predictor variables used in weighted resource selection functions for mainland gray wolves (*Canis lupus*), moose (*Alces alces*), and white-tailed deer (*Odocoileus virginianus*) on and near the Grand Portage Indian Reservation, Minnesota, USA, 2010–2021 and island wolves and moose on Isle Royale National Park, Michigan, USA, 2018–2021. The left panel column represents land cover as a categorical variable where open water was the reference category. The right panel column represents continuous predictor variables aspect (negative values represent south), slope, distance to nearest road (mainland only) or trail (island only), and distance to Lake Superior. Continuous predictor variables were considered significant if their confidence intervals did not overlap zero. The top three panel rows display seasonal selection, and the bottom panel row displays annual selection

Mainland space use states were characterized by species-specific season-specific resource selection. Mainland gray wolves (40 wolf-summers, 35 wolf-winters) selected for wetlands, shrublands, and deciduous forests during summer and for south-facing slopes during winter at the home range level (Fig. 4). Landscape variables did not predict core range resource selection, and step-lengths did not vary between space use states. Mainland moose (263 moose-summers, 228 moose-winters; spring and pre-parturition shifts were not analyzed because too few locations were available for analysis) selected for flatter slopes in core ranges year round and in summer home ranges. Within winter core ranges, mainland moose selected for shrublands, nearer roads, farther from Lake Superior, and south-facing slopes and had shorter step-lengths. Mainland white-tailed deer (90 deer-summers, 100 deer-winters) selected for shrublands in summer core ranges and deciduous forests in summer home ranges; deer selected for steeper slopes in winter home ranges and had shorter step-lengths. Landscape variables did not predict deer winter core range resource selection.

Island gray wolves and moose did not form stable seasonal space use state clusters, indicating space use did not change seasonally among these populations. Island wolves (27 wolf-years) did not select for any specific resource at the home range-level but selected for south-facing slopes and coniferous forests as well as against deciduous forests within their core ranges (Fig. 4). Island moose (86 moose-years) selected for south-facing slopes, shrublands, and coniferous forests as well as against wetlands and mixed forests at the home range-level. Within core ranges, island moose selected for south-facing slopes and against deciduous forests.

## Discussion

Supporting the ecological seasonality concept and our corresponding prediction, gray wolves altered their space use seasonally on the mainland where primary prey were migratory (i.e., white-tailed deer) but not on the island where primary prey were non-migratory (i.e., moose). Previous studies indicated similar gray wolf responses to migratory or non-migratory prey but did not compare against nearby areas with alternative prey movement strategies (Walton et al. 2001; Anderson 2014; Roffler et al. 2018). Adaptation of predators to prey migration occurs in multiple taxa. Spotted hyenas (*Crocuta crocuta*) exhibited space use changes in pursuit of several species of migratory prey (Trinkel et al. 2004), and grizzly bear (*Ursus arctos*) movements coincided with salmon (*Oncorhynchus* spp.) migrations (Glenn and Miller 1980). However, neither species’ responses were tested in systems with non-migratory prey.

Corresponding to the gray wolf response to space use by prey, we found that mainland and island wolves selected habitats also selected by prey. Mainland wolves selected for wetlands, shrublands, and deciduous forests in summer—which are selected by moose and white-tailed deer for foraging (Tremblay et al. 2005; Street et al. 2016)—and south-facing slopes in winter—which support better winter forage and are generally warmer with reduced snow levels (Pearson et al. 1995; Zweifel-Schielly et al. 2009). Island wolves did not exhibit resource selection within their home ranges reflecting similar use of available space but selected for south-facing slopes and coniferous forests within core ranges, habitats with better moose forage year round (Stephens and Peterson 1984; Zweifel‐Schielly et al. 2009). Our analysis does not, however, address the quality of habitats regarding predation success or risk (Sih 1998; Hebblewhite et al. 2005).

We found mixed support for our prediction that parturition would influence space use among gray wolves. Mainland wolves switched space use states immediately before parturition, but island wolves did not. Mainland wolves maintained similar space use patterns for > 6 months following parturition and did not exhibit movement constraints during denning. Wolves elsewhere altered their space and resource use multiple times during denning and rendezvous periods by decreasing movements and selecting flatter slopes (Ciucci and Mech 1992; Basille et al. 2013; Benson et al. 2015). Contrasting these studies, we used animal-defined seasons with longer thresholds (i.e., seasonal transition thresholds [8 days] greater than moving windows [5 days]).

Supporting our predictions, mainland moose, but not island moose, exhibited seasonal variation in space use, suggesting migration-altered space use extends to other species at the same trophic level. Supporting our interpretation, deer, moose, and beaver are primary prey of wolves in our study systems (Chenaux-Ibrahim 2015; Sovie et al. 2023), and despite beaver availability decreasing during winter due to seasonal freezing (Latham et al. 2013), we did not observe seasonal space use changes among island populations in the absence of deer. Further, wolves exhibited greater spatial overlap with moose in summer after spring deer migration (Wehr et al. 2024b), and wolf predation of moose and deer corresponded to their availability (Barber-Meyer and Mech 2016).

Space use among moose and white-tailed deer appeared to represent seasonal species-specific forage and cover needs. Mainland moose selected for flatter slopes nearer water in summer, needed for thermal refuge, escape terrain, and foraging (Stephens and Peterson 1984; McCann et al. 2016; Street et al. 2016), and for shrublands and nearer roads in winter, as observed elsewhere (Ball and Dahlgren 2002; Street et al. 2016). Deer selected for shrublands and deciduous forests in summer and steeper slopes in winter supporting foraging and mobility (Pearson et al. 1995; Tremblay et al. 2005; Zweifel-Schielly et al. 2009). Though seasonally unchanging, island moose resource selection may also be explained by forage and cover. Island moose browsing differs from mainland moose due to historic overbrowsing (Krefting 1974; Petz Giguere et al. 2024). Opposite to mainland moose, island moose selected for coniferous forests and against wetlands and deciduous forests, which might otherwise be expected (Stephens and Peterson 1984; Petz Giguere et al. 2024). Decreased forage availability may increase moose selection for conifers because they provide cover, mobility, and less-preferred forage during winter (Balsom et al. 1996).

Beyond our predictions, our results may provide additional insights into ecological seasonality and apparent competition among moose and white-tailed deer. Like gray wolves, mainland moose altered space use states concurrent to white-tailed deer migration. This change may occur because prey space use is influenced by predator–prey community dynamics via apparent competition as previously observed in moose–deer systems (Holt 1977; Forbes and Theberge 1996; Barber-Meyer and Mech 2016). Supporting this conclusion, mainland wolves increased spatial overlap with moose during summer when deer were less abundant after spring migration (Wehr et al. 2024b), which could produce a spatial response by moose as suggested for moose co-occurring with migratory caribou (Anderson 2014). This hypothesis warrants further examination as we were unable to find examples of reported migration-driven apparent competition altering space use. Satterfield et al. (2020) posited seasonal migratory insect arrival could alter resident insect behavior when predators switch to consuming the abundant migratory species, but offered no examples. Other scenarios support the possibility; for example, moose predation risk was greater during winter when caribou migrated to higher elevations (Seip 1992), but a space use response was not reported. The absence of moose space use changes in our island system also provides support for migration-driven apparent competition. However, the lack of moose space use changes could be consequent of simpler predator–prey dynamics as island moose do not face neonate predation pressure from black bears, coyotes (*Canis latrans*), or bobcats (*Lynx rufus*) (Sih 1998; Kautz et al. 2019; Wehr et al. 2023) or because the high island moose density increases intraspecific resource competition relative to mainland moose (Messier 1994; Romanski et al. 2020).

In summary, we observed differences in seasonal space use states between populations of predators and prey inhabiting ecosystems with differing mammal communities but otherwise similar characteristics. Mainland gray wolves and moose exhibited seasonal space use alterations concurrent to white-tailed deer migration while island wolves and moose allopatric with deer did not. Our results suggest plastic adaptation to site-specific community dynamics across trophic levels (Roffler et al. 2021) in support of the ecological seasonality concept (Basille et al. 2013). Our insights into ecological seasonality were limited to space use, and future studies could consider behavior, such as mate-seeking or diet, to improve temporal estimates of ecological seasons. Our results also support several aspects of predator–prey theory including alternative prey hypothesis (Lack 1954). Further, we observed an intraguild response of moose to deer migration, which may suggest apparent competition between these species (Holt 1977; Barber-Meyer and Mech 2016) and warrants study among other taxa.

## Data Availability

Data from the Grand Portage Indian Reservation used for this project has not been made publicly available to protect Indigenous data sovereignty; to request access, please contact the Grand Portage Band of Lake Superior Chippewa Department of Biology and Environment. Data from Isle Royale National Park may be accessed via application to the National Park Service.

## Code availability

Code used to generate results, cluster analysis decisions (including number of clusters considered, cluster stability, and seasonal transition dates), and means and effect sizes of weighted resource selection functions are available on Zenodo at 10.5281/zenodo.16763412.

## References

[CR1] Alston JM, Fleming CH, Kays R, Streicher JP, Downs CT, Ramesh T, Calabrese JM (2022) Mitigating pseudoreplication and bias in resource selection functions with autocorrelation-informed weighting. Methods Ecol Evol 14:643–654

[CR2] Anderson MS (2014) The role of human altered landscapes and predators in the spatial overlap between moose, wolves, and endangered caribou. Thesis, University of Alberta, Edmonton, Alberta, Canada

[CR3] Ball JP, Dahlgren J (2002) Browsing damage on pine (*Pinus sylvestris* and *P. contorta*) by a migrating moose (*Alces alces*) population in winter: relation to habitat composition and road barriers. Scand J For Res 17:427–435

[CR4] Balsom S, Ballard WB, Whitlaw HA (1996) Mature coniferous forest as critical moose habitat. Alces 32:131–140

[CR5] Barber-Meyer SM, Mech LD (2016) White-tailed deer (*Odocoileus virginianus*) subsidize gray wolves (*Canis lupus*) during a moose (*Alces americanus*) decline: a case of apparent competition? Can Field Naturalist 130:308–314

[CR6] Basille M, Fortin D, Dussault C, Ouellet JP, Courtois R (2013) Ecologically based definition of seasons clarifies predator–prey interactions. Ecography 36:220–229

[CR7] Benson JF, Mills KJ, Patterson BR (2015) Resource selection by wolves at dens and rendezvous sites in Algonquin park, Canada. Biol Conserv 182:223–232

[CR8] Berger J (2007) Fear, human shields and the redistribution of prey and predators in protected areas. Biol Lett 3:620–623 17925272 10.1098/rsbl.2007.0415 PMC2391231

[CR9] Brown JS, Laundré JW, Gurung M (1999) The ecology of fear: optimal foraging, game theory, and trophic interactions. J Mammal 80:385–399

[CR10] Carmone FJ Jr, Kara A, Maxwell S (1999) HINoV: a new model to improve market segment definition by identifying noisy variables. J Mark Res 36:501–509

[CR11] CCRS CCMEO, NRCan CONABIO, INEGI CONAFOR, USGS (2020) 2015 land cover of North America at 30 meters. Canada Centre for Remote Sensing, Ottawa, Ontario, Canada

[CR12] Chenaux-Ibrahim Y (2015) Seasonal diet composition of gray wolves (*Canis lupus*) in northeastern Minnesota determined by scat analysis. Thesis, University of Minnesota, Minneapolis, Minnesota, USA

[CR13] Ciucci P, Mech LD (1992) Selection of wolf dens in relation to winter territories in northeastern Minnesota. J Mammal 73:899–905

[CR14] Corkeron PJ, Connor RC (1999) Why do baleen whales migrate? Mar Mamm Sci 15:1228–1245

[CR15] Darlington S, Ladle A, Burton AC, Volpe JP, Fisher JY (2022) Cumulative effects of human footprint, natural features and predation risk best predict seasonal resource selection by white-tailed deer. Sci Rep 12:1072 35058533 10.1038/s41598-022-05018-z PMC8776810

[CR16] de Amorim RC, Hennig C (2015) Recovering the number of clusters in data sets with noise features using feature rescaling factors. Inf Sci 324:126–145

[CR17] Dormann CF, Elith J, Bacher S, Buchmann C, Carl G, Carré G, Marquéz JRG, Gruber B, Lafourcade B, Leitao PJ (2013) Collinearity: a review of methods to deal with it and a simulation study evaluating their performance. Ecography 36:27–46

[CR18] Fleming CH, Calabrese JM (2023) ctmm: continuous-time movement modeling. R package version 1.2.0

[CR19] Forbes G, Theberge J (1996) Response by wolves to prey variation in central Ontario. Can J Zool 74:1511–1520

[CR20] Fox J (2015) Applied regression analysis and generalized linear models. Sage, Thousand Oaks, California, USA

[CR21] Frades I, Matthiesen R (2010) Overview on techniques in cluster analysis. In: Matthiesen R (ed) Bioinformatics methods in clinical research. Humana, New York, New York, USA, pp 81–107 10.1007/978-1-60327-194-3_5 19957146

[CR22] Furey NB, Armstrong JB, Beauchamp DA, Hinch SG (2018) Migratory coupling between predators and prey. Nat Ecol Evol 2:1846–1853 30467414 10.1038/s41559-018-0711-3

[CR23] Gilbert JH, David P, Price MW, Oren J (2022) Ojibwe perspectives toward proper wolf stewardship and Wisconsin’s February 2021 wolf hunting season. Front Ecol Evol 10:782840

[CR24] Gill R, Serrouya R, Calvert AM, Ford A, Steenweg R, Noonan MJ (2023) Movement ecology of endangered caribou during a COVID-19 mediated pause in winter recreation. Anim Conserv 27:350–363

[CR25] Glenn LP, Miller LH (1980) Seasonal movements of an Alaska Peninsula brown bear population. International Conference on Bear Research and Management 4:307–312

[CR26] Hebblewhite M, Merrill EH, McDonald TL (2005) Spatial decomposition of predation risk using resource selection functions: an example in a wolf–elk predator–prey system. Oikos 111:101–111

[CR27] Hennig C (2007) Cluster-wise assessment of cluster stability. Comput Stat Data Anal 52:258–271

[CR28] Hennig C (2020) fpc: flexible procedures for clustering. R package version 2.2-9

[CR29] Holt RD (1977) Predation, apparent competition, and the structure of prey communities. Theor Popul Biol 12:197–229 929457 10.1016/0040-5809(77)90042-9

[CR30] Hoy SR, Peterson RO, Vucetich JA (2019) Ecological studies of wolves on Isle Royale, 2018–2019. Technical Report, Michigan Technological University, Houghton, Michigan, USA

[CR31] Ienello L, Moore S, Isaac EJ, Thompson R, Guedes AGP, Wolf TM (2025) Physiologic outcomes after thiafentanil and xylazine immobilization in free-ranging moose (*Alces alces*). J Wildl Dis 61:247–252 39573904 10.7589/JWD-D-24-00114

[CR32] Kassambra A (2023) ggpubr: ‘ggplot2’ based publication ready plots. R package version 0.6.0

[CR33] Kassambra A, Mundt F (2020) factoextra: extract and visualize the results of multivariate data analyses. R package version 1.0.7. https://CRAN.R-project.org/package=factoextra

[CR34] Kautz TM, Belant JL, Beyer DE Jr, Strickland BK, Petroelje TR, Sollmann R (2019) Predator densities and white-tailed deer fawn survival. J Wildl Manage 83:1261–1270

[CR35] Krefting LW (1974) The ecology of the Isle Royale moose with special reference to the habitat. University of Minnesota, Minneapolis, Minnesota, USA

[CR36] Lack D (1954) Cyclic mortality. J Wildl Manage 18:25–37

[CR37] Latham ADM, Latham MC, Knopff KH, Hebblewhite M, Boutin S (2013) Wolves, white-tailed deer, and beaver: implications of seasonal prey switching for woodland caribou declines. Ecography 36:1276–1290

[CR38] Mayor SJ, Schneider DC, Schaefer JA, Mahoney SP (2009) Habitat selection at multiple scales. Ecoscience 16:238–247

[CR39] McCann NP, Moen RA, Windels SK, Harris TR (2016) Bed sites as thermal refuges for a cold-adapted ungulate in summer. Wildl Biology 22:228–237

[CR40] Messier F (1994) Ungulate population models with predation: a case study with the North American moose. Ecology 75:478–488

[CR41] Mitchell WA, Lima SL (2002) Predator-prey shell games: large‐scale movement and its implications for decision‐making by prey. Oikos 99:249–259

[CR42] Moore SA, Wolf TM, Severud WJ, Isaac EJ, Chenaux-Ibrahim YM (2024) Spring black bear harvest and predation pressure on moose calves in a multi-predator system. J Wildl Manage 88:e22618

[CR43] Moorhouse-Gann RJ, Kean EF, Parry G, Valladares S, Chadwick EA (2020) Dietary complexity and hidden costs of prey switching in a generalist top predator. Ecol Evol 10:6395–6408 32724521 10.1002/ece3.6375 PMC7381573

[CR44] Muff S, Signer J, Fieberg J (2020) Accounting for individual-specific variation in habitat‐selection studies: efficient estimation of mixed‐effects models using Bayesian or frequentist computation. J Anim Ecol 89:80–92 31454066 10.1111/1365-2656.13087

[CR46] NOAA (2024) Climate Data Online (CDO). National Oceanic and Atmospheric Administration, Asheville, North Carolina, USA

[CR45] NOAA (2000) NOAA medium resolution shoreline. National Oceanic and Atmospheric Administration, Washington, D.C., United States

[CR47] Northrup JM, Anderson CR Jr, Wittemyer G (2014) Effects of helicopter capture and handling on movement behavior of mule deer. J Wildl Manage 78:731–738

[CR48] O’Brien O, Allen S, Krützen M, Connor R (2020) Alliance-specific habitat selection by male Indo-Pacific bottlenose dolphins in Shark Bay, Western Australia. Anim Behav 164:39–49

[CR49] Oliveira-Santos LGR, Moore SA, Severud WJ, Forester JD, Isaac EJ, Chenaux-Ibrahim Y, Garwood T, Escobar LE, Wolf TM (2021) Spatial compartmentalization: a nonlethal predator mechanism to reduce parasite transmission between prey species. Sci Adv 7:eabj5944 34936450 10.1126/sciadv.abj5944 PMC8694586

[CR50] OMNRF (2022) Ontario Road Network (ORN) segnment with address. Ontario Ministry of Natural Resources and Forestry, Peterborough, Ontario, Canada

[CR51] Orning EK, Romanski MC, Moore SA, Chenaux-Ibrahim Y, Hart J, Belant JL (2020) Emigration and first-year movements of initial wolf translocations to Isle Royale. Northeastern Naturalist 27:701–708

[CR52] Pearson SM, Turner MG, Wallace LL, Romme WH (1995) Winter habitat use by large ungulates following fire in northern Yellowstone National Park. Ecol Appl 5:744–755

[CR53] Petz Giguere MT, Severud WJ, Teager K, Wolf TM, Moore SA (2024) Twig production patterns among moose forage species and implications for forest management. Alces 60:75–108

[CR54] R Core Team (2025) R: a language and environment for statistical computing. R Foundation for Statistical Computing, Vienna, Austria

[CR56] Roffler GH, Gregovich DP (2018) Wolf space use during denning season on Prince of Wales Island, Alaska. Wildlife Biology 2018:wlb.00468

[CR57] Roffler GH, Gregovich DP, Larson KR (2018) Resource selection by coastal wolves reveals the seasonal importance of seral forest and suitable prey habitat. For Ecol Manag 409:190–201

[CR55] Roffler GH, Allen JM, Massey A, Levi T (2021) Metabarcoding of fecal DNA shows dietary diversification in wolves substitutes for ungulates in an island archipelago. Ecosphere 12:e03297

[CR58] Romanski MC, Orning EK, Kellner KF, Beyer DE Jr, Brzeski KE, Hart J, Lonsway DH Jr, McLaren AAD, Moore SA, Patterson BR et al (2020) Wolves and the Isle Royale environment: restoring an island ecosystem, 2018–2020. Technical Report, National Park Service, Isle Royale National Park, Michigan, USA

[CR59] Satterfield DA, Sillett TS, Chapman JW, Altizer S, Marra PP (2020) Seasonal insect migrations: massive, influential, and overlooked. Front Ecol Environ 18:335–344

[CR60] Schooler SL, Finnegan SP, Fowler NL, Kellner KF, Lutto AL, Parchizadeh J, van den Bosch M, Zubiria Perez A, Masinde LM, Mwampeta SB et al (2022) Factors influencing lion movements and habitat use in the western Serengeti ecosystem. Tanzan Sci Rep 12:18890 10.1038/s41598-022-22053-y PMC9640537 36344560

[CR61] Seip DR (1992) Factors limiting woodland caribou populations and their interrelationships with wolves and moose in southeastern British Columbia. Can J Zool 70:1494–1503

[CR62] Signer J, Fieberg J, Avgar T (2019) Animal movement tools (amt): R package for managing tracking data and conducting habitat selection analyses. Ecol Evol 9:880–890 30766677 10.1002/ece3.4823 PMC6362447

[CR63] Sih A (1998) Game theory and predator–prey response races. In: Dugatkin L, Reeve H (eds) Game theory and animal behavior. Oxford University Press, Oxford, United Kingdom, pp 221–238

[CR64] Sinclair EH, Zeppelin TK (2002) Seasonal and spatial differences in diet in the western stock of Steller sea lions (*Eumetopias jubatus*). J Mammal 83:973–990

[CR65] Smereka CA, Frame PF, Edwards MA, Frame DD, Slater OM, Derocher AE (2020) Seasonal habitat selection of cougars *Puma concolor* by sex and reproductive state in west-central Alberta, Canada. Wildlife Biology 2020:wlb.00735

[CR66] Sovie AR, Romanski MC, Orning EK, Marneweck DG, Nichols R, Moore SA, Belant JL (2023) Temporal variation in translocated Isle Royale wolf diet. Ecol Evol 13:e9873 36937055 10.1002/ece3.9873 PMC10019911

[CR67] Stephens PW, Peterson RO (1984) Wolf-avoidance strategies of moose. Ecography 7:239–244

[CR68] Street GM, Fieberg J, Rodgers AR, Carstensen M, Moen R, Moore SA, Windels SK, Forester JD (2016) Habitat functional response mitigates reduced foraging opportunity: implications for animal fitness and space use. Landscape Ecol 31:1939–1953

[CR69] Theon E (2022) padr: quickly get datetime data ready for analysis. R package version 0.6.2

[CR70] Thompson DP (2020) The right to hunt and fish therein: understanding Chippewa treaty rights in Minnesota’s 1854 Ceded Territory. 1854 Treaty Authority, Duluth, Minnesota, USA

[CR71] Tischler KB, Severud WJ, Peterson RO, Vucetich JA, Bump JK (2022) Aquatic areas provide high nitrogen forage for moose (*Alces alces*) in Isle Royale National Park, Michigan, USA. Alces 58:75–90

[CR72] Tremblay JP, Thibault I, Dussault C, Huot J, Côté SD (2005) Long-term decline in white-tailed deer browse supply: can lichens and litterfall act as alternative food sources that preclude density-dependent feedbacks. Can J Zool 83:1087–1096

[CR73] Trinkel M, Fleischmann PH, Steindorfer AF, Kastberger G (2004) Spotted hyenas (*Crocuta crocuta*) follow migratory prey. Seasonal expansion of a clan territory in Etosha, Namibia. J Zool 264:125–133

[CR74] Tschanz B, Bersier LF, Bacher S (2007) Functional responses: a question of alternative prey and predator density. Ecology 88:1300–1308 17536415 10.1890/06-1512

[CR75] USCB (2021) TIGER data products. U.S. Department of Commerce, Suitland, Maryland, USA

[CR76] USGS (2020) 1 arc-second digital elevation models (DEMs) - USGS National Map 3DEP dowloadable data collection. United States Geological Survey, Reston, Virginia, USA

[CR77] van Wijk RE, Kölzsch A, Kruckenberg H, Ebbinge BS, Müskens GJDM, Nolet BA (2012) Individually tracked geese follow peaks of temperature acceleration during spring migration. Oikos 121:655–664

[CR78] Viechtbauer W (2010) Conducting meta-analyses in R with the metafor package. J Stat Softw 36:1–48

[CR79] Walesiak M, Dudek A (2021) Searching for optimal clustering procedure for a data set. R package version 0.49-2

[CR80] Walton LR, Cluff HD, Paquet PC, Ramsay MA (2001) Movement patterns of barren-ground wolves in the central Canadian Arctic. J Mammal 82:867–876

[CR81] Wehr NH (2023) Seasonal movement, space use, and mortality of gray wolves, moose, and white-tailed deer in northeastern Minnesota. Dissertation, Michigan State University, East Lansing, Michigan, USA

[CR82] Wehr NH, Boone HM, Wehr SR, Belant JL (2023) Island characteristics and species traits predict mammal diversity across islands of the great lakes of North America. Biodivers Conserv 32:3465–3480

[CR83] Wehr NH, Moore SA, Isaac EJ, Kellner KF, Millspaugh JJ, Belant JL (2024a) Moose and white-tailed deer mortality peaks in fall and late winter. J Wildl Manage 88:e22580

[CR84] Wehr NH, Moore SA, Isaac EJ, Kellner KF, Millspaugh JJ, Belant JL (2024b) Spatial overlap of gray wolves and ungulate prey changes seasonally corresponding to prey migration. Mov Ecol 12:33 38671527 10.1186/s40462-024-00466-w PMC11046751

[CR85] Werner EE, Hall DJ (1974) Optimal foraging and the size selection of prey by the bluegill sunfish (*Lepomis macrochirus*). Ecology 55:1042–1052

[CR86] Zweifel-Schielly B, Kreuzer M, Ewald KC, Suter W (2009) Habitat selection by an Alpine ungulate: the significance of forage characteristics varies with scale and season. Ecography 32:103–113

